# Substitution rate heterogeneity across hexanucleotide contexts in noncoding chloroplast DNA

**DOI:** 10.1093/g3journal/jkac150

**Published:** 2022-06-14

**Authors:** Brian R Morton

**Affiliations:** Department of Biology, Barnard College, Columbia University, New York, NY 10027, USA

**Keywords:** chloroplast DNA, mutation, context, genome evolution, substitution model

## Abstract

Substitutions between closely related noncoding chloroplast DNA sequences are studied with respect to the composition of the 3 bases on each side of the substitution, that is the hexanucleotide context. There is about 100-fold variation in rate, among the contexts, particularly on substitutions of A and T. Rate heterogeneity of transitions differs from that of transversions, resulting in a more than 200-fold variation in the transitions: transversion bias. The data are consistent with a CpG effect, and it is shown that both the A + T content and the arrangement of purines/pyrimidines along the same DNA strand are correlated with rate variation. Expected equilibrium A + T content ranges from 36.4% to 82.8% across contexts, while G–C skew ranges from −77.4 to 72.2 and A–T skew ranges from −63.9 to 68.2. The predicted equilibria are associated with specific features of the content of the hexanucleotide context, and also show close agreement with the observed context-dependent compositions. Finally, by controlling for the content of nucleotides closer to the substitution site, it is shown that both the third and fourth nucleotide removed on each side of the substitution directly influence substitution dynamics at that site. Overall, the results demonstrate that noncoding sites in different contexts are evolving along very different evolutionary trajectories and that substitution dynamics are far more complex than typically assumed. This has important implications for a number of types of sequence analysis, particularly analyses of natural selection, and the context-dependent substitution matrices developed here can be applied in future analyses.

## Introduction

A relationship between mutation rate heterogeneity and neighboring base composition, or context, has been observed across a wide range of taxa in studies of both de novo mutations (Ossowski *et al.* 2010; Michaelson *et al.* 2012; Sung *et al.* 2015; Francioli *et al.* 2015; Smith *et al.* 2018) and single-nucleotide variant (SNV) data (Morton *et al.* 2006; Aggarwala and Voight 2016; Zhu *et al.* 2017; Simon and Huttley 2020). In many cases, the data show that context has a fairly complex relationship with mutations. For example, in the human genome, variation in composition of the 6 nucleotides, 3 on each side, surrounding a mutation site, accounts for most mutational heterogeneity (Aggarwala and Voight 2016), with variation in the rate of transversions being influenced by a slightly larger context than is variation in transitions (Zhu *et al.* 2017; Simon and Huttley 2020) and in *Escherichia* *coli* mutation rate varied about 75-fold across contexts (Sung *et al.* 2015). Since heterogeneity in mutation dynamics across sites can have a significant influence on various analyses, particularly analyses of selection (Morton 2003; Sung *et al.* 2015) as recently demonstrated for selection on codon usage in chloroplast genes (Morton 2022a), it is important to gain a better understanding of the context-dependent nature of mutations.

Probably, the most obvious context effect on mutation rate is the hypermutability of methylated cytosines, such as what is observed at CpG sites in mammals (Ehrlich and Wang 1981; Arndt *et al.* 2003; Huttley 2004; Mugal and Ellegren 2011; Francioli *et al.* 2015; Simon and Huttley 2020). However, while the CpG effect, which is the result of a high rate of deamination of methylated cytosines (Coulondre *et al.* 1978), plays a notable role, the often complex relationship between context and mutation has made it clear that additional factors must be involved. These could include an influence of context on polymerase misincorporation (Mendelman *et al.* 1989; Aggarwala and Voight 2016), on mismatch repair (Kunkel and Erie 2015; Burgers and Kunkel 2017), which might depend on local helix stability due to base stacking properties (SantaLucia and Hicks 2004; Ghosh *et al.* 2019; Zacharias 2020) but just how these, or additional, factors actually affect mutation rate heterogeneity remains unclear.

A relationship between context, particularly the A + T content of flanking bases, and substitution dynamics, particularly the ratio of transitions:transversions (Ts:Tv), at putatively neutral sites has long been known to exist in angiosperm chloroplast DNA, or cpDNA (Morton 1995; Morton and Clegg 1995; Zheng *et al.* 2007). Early studies showed that the A + T content of nucleotides flanking a substitution is correlated with the Ts:Tv bias, and recently, it has been shown that such context effects are observed in all major plastid lineages and can include an influence of nucleotides up to 4 sites removed from the substitution (Morton 2022b).

Due to data availability, previous analyses of cpDNA focused on the general context features and substitution dynamics noted above. The current analysis takes advantage of the large number of angiosperm chloroplast complete genome sequences now available to study the full range of local contexts within 4 bases on each side of the substitution and the full spectrum of substitution types. The low evolutionary rate of cpDNA (Clegg 1993) results in a dearth of SNV data but allows for comparisons of highly similar sequence pairs to study substitution dynamics at putatively neutral sites, with an outgroup taxon used to infer substitution direction. Here, 280 genome triplets yielded 1,001,783 substitutions within intergenic (NC) regions. Each substitution was analyzed as the central site of a heptanucleotide, providing evidence about the surrounding hexanucleotide context. These substitutions were used to generate 4 × 4 substitution matrices, which allow for an analysis of rate heterogeneity across contexts as well as more general heterogeneity in substitution dynamics by comparing expected equilibrium base composition across contexts when each substitution matrix is converted to a transition matrix.

Across the 4,096 hexanucleotide contexts, roughly 100-fold variation in rate is observed, with greater heterogeneity observed in the rate of transversions than of transitions. There is clear evidence for a CpG effect, but other context features are also found to be associated with rate variation, in particular the A + T content and the R/Y arrangement along 1 strand. Controlling for the composition of the first 2 nucleotides flanking substitutions, it is shown that the third and fourth nucleotides removed from substitutions directly influence substitution rate, particularly the rate of transversions. In addition to measurements of rate, there is also a great deal of heterogeneity in the equilibrium base composition predicted for each hexanucleotide context. The expected equilibrium A + T content ranges from 36.4% to 82.8%, while predicted skew ranges from −77.4 to 72.2 for G–C, and from −63.9 to 68.2 for A–T. The heterogeneity in compositional equilibria shows that context effects in cpDNA have tremendous importance for sequence evolution and for analyses that rely on substitution or mutation models. Neutral sites cannot be compared in a context-independent manner given the widely divergent evolutionary trajectories. Most critically, models that account for this, and evolving context at the same time, will be needed for more accurate sequence analysis and the context-dependent models developed here can be utilized in this way.

## Materials and methods

RefSeq complete angiosperm chloroplast genome sequences were downloaded from NCBI (www.ncbi.nlm.nih.gov/genome/browse#!/eukaryotes/) on March 14, 2019, and then parsed with the Biopython 1.76 (Cock *et al.* 2009). Regions between neighboring Biopython SeqRecords were saved as intergenic (NC) regions. Genomes were grouped into 280 closely related triplets from 40 different families (listed Morton 2022a) which were chosen to ensure nonoverlapping lineages, and each NC region greater than 70 nucleotides in length was then aligned as previously described (Morton 2022a).

The alignments were used to 4,096 hexanucleotide context-dependent substitution matrices, 1 matrix for each hexanucleotide context. Any site N_0_ can be considered as the central site of the heptanucleotide N_3_ N_2_ N_1_ [N_0_] N_1_ N_2_ N_3_ where N_1_, N_2_, and N_3_ are the neighboring base pairs flanking N_0_. In each context-dependent matrix CM, CM_*i*__,__*j*_ represents the number of sites at which there was an ancestral nucleotide *i* and derived nucleotide *j*, indicating an *i* → *j* substitution. For every aligned site within a conserved hexanucleotide context in all 3 sequences, CM_*a*__,__*s1*_ and CM_*a*__,__*s2*_ are incremented by 1, where *a* is the ancestral nucleotide inferred from the outgroup, s1 and s2 are the nucleotides in the 2 ingroup sequences. Conserved sites were included and provide diagonal values for the matrices. To exclude comparisons of nonhomologous regions within alignments without biasing inferences of context effects, the restrictions described in Morton (2022a) were followed. For a site to be included in the analysis, at least 8 of the 10 surrounding alignment sites had to be base pairs in both ingroup sequences (i.e. no gap in either sequence) and the 2 ingroup sequences needed to have at least 70% similarity across these 10 sites.

Sample size within each context was maximized by combining complementary matrix pairs (e.g. the AGC[N]TGA context with the complement of the TCA[N]GCT context) as in Morton (2022a). This reduces the number of unique hexanucleotide contexts to 2,080 for any analysis that considers only one of each pair of complementary matrices, such as AAA[N]AA and TTT[N]TTT. Since the context of each conserved site was also recorded, a Markov transition matrix Π can be generated from any substitution matrix, CM, by setting Π_*i*__,__*j*_ = CM_*i*__,__*j*_/ΣCM_*i*_. These matrices can also be taken as estimates of substitution rate with the units substitutions/site per average time of all branches in the ingroup taxa, and so the values will be referred to as rates, each with a confidence interval equal to 1.96 $\sqrt{p\left(1-p\right)n}$ where *n* is the row sum.

For each context-dependent matrix Π, the stationary vector ϕ was calculated such that ϕ = ϕΠ, which represents the equilibrium base frequency for a site evolving with the mutation dynamics given by Π (Cox and Miller 2017; Morton 2022a). For these analyses, only 1 matrix of each complementary pair was included since the second is redundant as a result of the combination of complementary matrices. All calculations were done using Python script written by the author.

Three summary statistics of context composition were defined and calculated for each hexanucleotide: the A + T Index (ATI), the Purine Index (RI), and the Purine A + T Index (RATI), which combines the 2 other features. Each statistic weights the A + T and/or Purine content on 1 strand as a function of distance from the substitution site. The RI value was used for analyses of substitutions of pyrimidines on that strand, which is redundant with using a Pyrimidine Index for analyses of substitutions of purines. These indexes were calculated as in the following equations:

(1) $$\mathrm{ATI}=\;\sum_{i=1}^{3}\mathrm{Wi}\times \;2^{\left(3-i\right)}$$

(2) $$\mathrm{RI}=\;\sum_{i=1}^{3}\mathrm{Ri}\times \;2^{\left(3-i\right)}$$

(3) $$\mathrm{RATI}=\;\sum_{i=1}^{3}\mathrm{Bi}\times \;2^{\left(3-i\right)}$$

In Equation (1), Wi is the number of AT base pairs in the N_i_ neighboring pair; in Equation (2), Ri is the number of purines on the strand analyzed in the N_i_ neighboring pair; and in Equation (3), Bi is the sum of the RATI values of the 2 bases in the N_i_ neighboring pair, with the RATI values defined as A = 2, C = 0, G = 1, and T = 1.

To test for strand symmetry, the genes *rps12*, *psbA*, *psbK*, *psbD*, and *psbC* were used as a reference set to determine strand equivalency since they are annotated in almost every genome, with the most common arrangement being that the first 2 genes are coded on 1 DNA strand and the latter 3 coded on the opposite strand. Each genome with all 5 reference genes annotated with this strand arrangement was retained (the symmetry genomes) and the orientation of each symmetry genome was set such that the strand with *rps12* and *psbA* was designated strand A and the other as strand C, regardless of NCBI annotation. Next, a list of all genes annotated in at least 90% the symmetry genomes was generated and, for each of these genes, the coding strand within each symmetry genome was determined based on the NCBI annotation. Finally, a list of the genes with a consistent strand arrangement relative to the 5 reference genes in at least 80% of the symmetry genomes, the symmetry genes, was generated (i.e. if gene “X” was annotated on the same strand as the 5 reference genes in either more than 80% or fewer than 20% of the genomes, then it was included). For the 152 genome triplets with all 3 genomes in the symmetry genome list, an alignment was generated using the strand A sequence for each intergenic region located between 2 genes on the symmetry gene list. From these alignments, context-dependent substitution matrices were generated from the entire set of alignments as described above. This approach will generate matrices of substitution data from the same DNA strand in every genome at the cost of excluding a lot of the sequence data. These matrices will be referred to as the Strand Symmetry Test Matrices.

## Results and discussion

Sites were scored with respect to their hexanucleotide contexts to generate 4,096 context-dependent substitution matrices, with conserved sites making up the matrix diagonals. There were 1,001,783 substitutions across 15,123,150 sites, which averages to 244.6 substitutions/matrix and 0.066 substitutions/site. Given the low substitutions per site, multiple hits will not be considered and substitutions/site will be referred to as the rate of substitution. Once complementary matrices were combined, as described in the *Materials and* *Methods*, there was a total of 1,889,456 substitutions at 29,187,360 sites, so the 4,096 context-dependent matrices average 465.8 substitutions, although complementary matrices become redundant in some analyses. As indicated below in these cases, only one of each complementary pair is included.

Some bacterial genomes show a pattern of skew centered on the replication origin (Lobry and Louarn 2003; Morton and Morton 2007) and the data here are from the annotated strand of each NCBI file. Although plant chloroplast genomes appear to use a double D-loop replication system (Morley *et al.* 2019) and do not display a skew pattern like bacteria, the matrices were tested for evidence of asymmetry using the Strand Symmetry Test Matrices generated from a subset of genomes (see *Materials and* *Methods*). Complementary contexts in the Strand Symmetry Test Matrices were compared and showed the same trends of heterogeneity across contexts. Predicted equilibrium A + T content on the 2 strands showed a correlation of *r*^2^ = 0.8717 across contexts with at least 50 substitutions, and for A–T skew, the correlation was *r*^2^ = 0.8921. Therefore, it was assumed that context effects are not strand asymmetric and so the larger dataset consisting of the combined complementary matrices from the original set of alignments was utilized in all further analyses.

### Rate variation across contexts

Of the hexanucleotide contexts with at least 50 transitions and 50 transversions, those with the highest and lowest of each of (1) overall substitution rate, (2) transition (Ts) rate, and (3) transversion (Tv) rate are shown in Supplementary Table S1. Variation is given separately for substitutions of T and substitutions of C. Since complementary matrices were combined as described above, the variation data for A and G are redundant and so we will only consider changes from T and C from hereon, which allows all 4,096 matrices (contexts) to be included. The fold differences between the contexts with the highest and lowest rates from C and T are summarized in Table 1 for contexts with at least 50 transitions and 50 transversions from the base analyzed (C or T) and for contexts with at least 100 substitutions from the base being analyzed (C or T). Substantial variation is seen across contexts, in some cases more than 100-fold. Both overall rate and Ts:Tv show greater variation across contexts for substitutions from T than for substitutions from C, and the variation in Tv rate across contexts is greater than variation in Ts rate, although there is particularly high variation in Tv rate in substitutions from T. The Ts and Tv rate variation yields a very wide range in Ts:Tv, which varies more than 200-fold across contexts from 0.12 (context CGA[T]AAT) to 27.5 (context ACC[T]CCT).

**Table 1.** Degree of variation in substitution rate across 4096 hexanucleotide contexts.

	Ts rate variation	Tv rate variation	Total rate variation	Ts:Tv variation
Ts > 50 and Tv > 50^a^				
From T	24.5^b^	44.6	21.0	36.4
From C	15.4	15.9	10.2	46.5
From T and C	31.4	44.6	25.1	60.8
(Ts + Tv) > 100^c^				
From T	49.9	109.5	21.0	229.2
From C	24.0	33.4	12.7	61.8
From T and C	61.7	109.5	25.1	229.2

a Only contexts that have at least 50 transitions and at least 50 transversions in that matrix row (from T or from C) are included.

b Values are given as the context with the highest rate divided by the context with the lowest rate.

c Only contexts that have at least 100 substitutions in that matrix row (from T or from C) are included.

### Context features that are associated with rate heterogeneity

There are 2 obvious composition features of the hexanucleotide contexts that are associated with the rate variation summarized in Table 1. One feature is the A + T content of the neighboring pairs, which has been shown previously to be associated with substitution bias in cpDNA (Morton 1995; Morton and Clegg 1995) and the other is the arrangement of purines and pyrimidines along 1 strand, a feature that has been shown to be associated with variation in mutation rate in bacteria (Sung *et al.* 2015). The contexts in Supplementary Table 1 with the lowest Ts rates have a greater average A + T content than do those with the highest Ts rates while the opposite association is seen for Tv rate (see Supplementary Table 1). This results in the association between Ts:Tv and flanking base A + T content that has been described previously (Morton 1995; Morton and Clegg 1995; Zheng *et al.* 2007; Morton 2022a). In addition, R[Y]R contexts, where the [Y] is the T or C at the substitution site, are more common in the higher rate contexts, particularly for substitutions of C, suggesting that the arrangement of purines and pyrimidines is a factor. Substitutions of Y in an at R[Y]R context also have lower Ts:Tv values, particularly for substitutions of T (see Supplementary Table S1). As a result, for example, a T flanked by 2 Gs on 1 strand (G[T]G = R[Y]R) is more likely to undergo a transversion to yield an RRR sequence than to undergo a transition, which would retain the RYR structure.

**Fig. 1.**
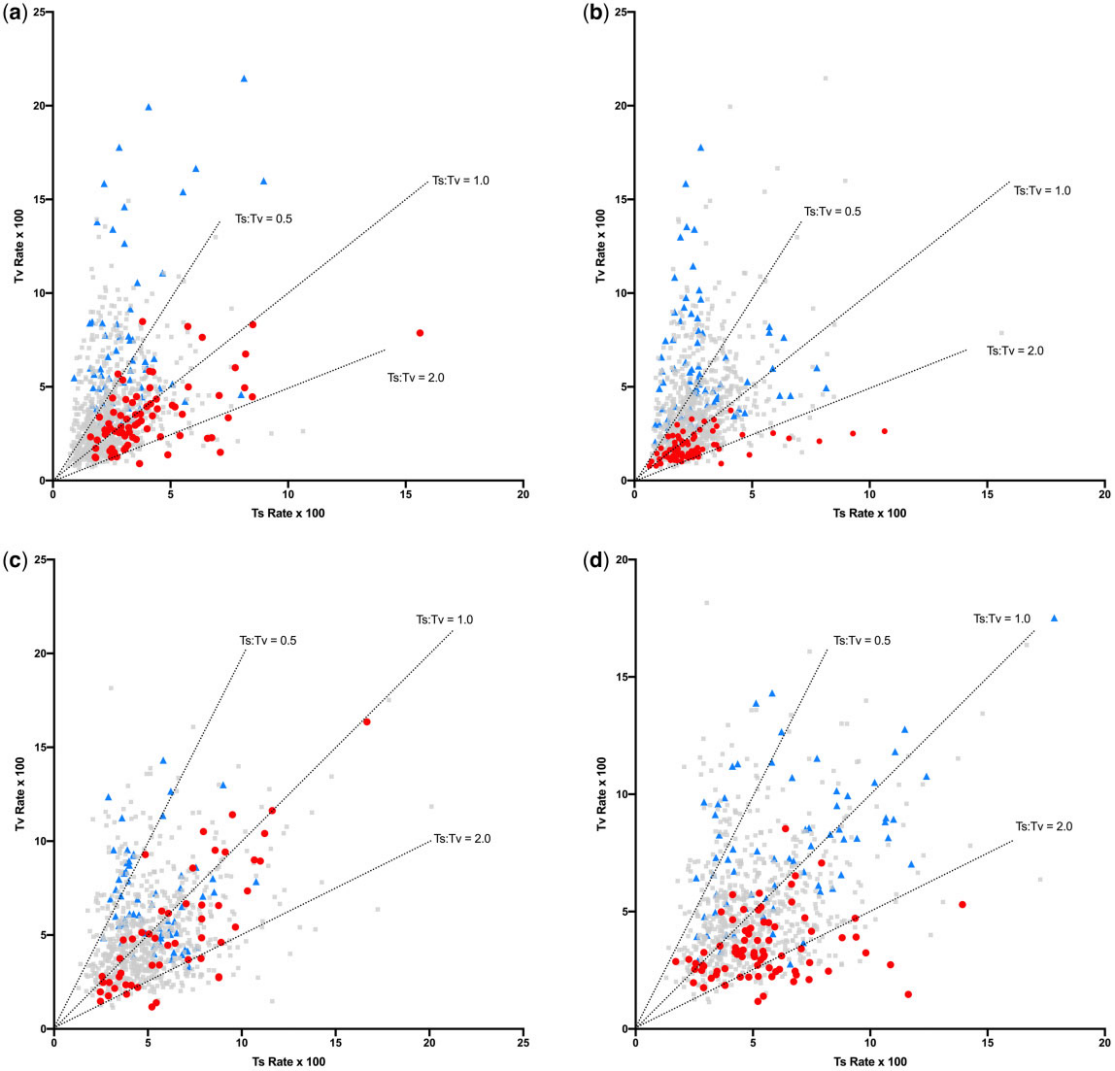
Transition and transversion rates for substitutions from T (a and b) and C (c and d) across hexanucleotide contexts in which there are at least 50 transitions and 50 transversions. In (a) and (c), contexts with an ATI of ≤12 are in red (circles) and those with ATI = 28 are in blue (triangles). In (b) and (d), contexts with an RI of ≤2 are in red and those with an RI of ≥26 are in blue. These cutoff values were chosen simply to highlight contexts at the 2 ends of these content ranges. Lines represent Ts:Tv values as indicated.

**Fig. 2.**
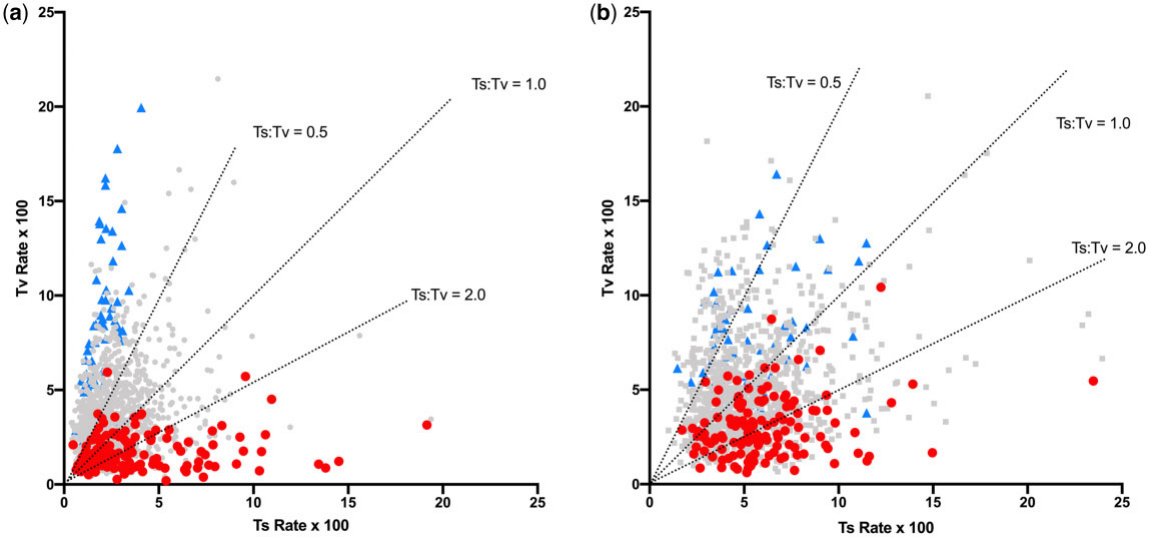
Transition and transversion rates for substitutions from T (a) and C (b) across hexanucleotide contexts in which there are at least 50 transitions and 50 transversions. Contexts with an RATI of <6 are in red (circles) and those with an ATI of >22 are in blue (triangles). These cutoff values were chosen simply to highlight contexts at the 2 ends of these content ranges. Lines represent Ts:Tv values as indicated.

Ts and Tv rates within all contexts are shown in Fig. 1, highlighting the influence of both A + T and Purine content of flanking bases just described. (A plot of these rates with 95% CI values is shown in Supplementary Fig. 1.) Substitutions from C and T have different general patterns. There is a generally higher substitution rate from C, with an average of 0.106 subs/site, than from T, with an average of 0.066 subs/site, which is also apparent in the distances from the plot origins. In addition, there is a generally higher Ts:Tv of substitutions from C, with an average across contexts from C and T of 1.17 and 0.89, respectively. For substitutions of T, sites in contexts with a high ATI have a relatively high Tv rate as do contexts with a high RI (Fig. 1, a and c), resulting in a lower Ts:Tv in these contexts. A similar but less pronounced trend is seen for substitutions from C (Fig. 1, b and d). The combined effect of the A + T and R context features, measured by RATI as described in the *Materials and* *Methods*, is illustrated in Fig. 2. Contexts with higher RATI values show much more variation in Tv rates, particularly for substitutions of T, while those with lower RATI values show more variation in Ts rates and, as a result, generally lower Ts:Tv values. Although Figs. 1 and 2 highlight the ends of the context composition ranges, a strong decrease in Ts:Tv, particularly in substitutions from T, is observed across the full range of all 3 context composition measures (Supplementary Fig. 2).

To explore the possibility that local base stacking properties (SantaLucia and Hicks 2004; Zacharias 2020) contribute to rate variation across contexts, the stability (ΔG_37_) of each heptanucleotide (the hexanucleotide context plus the internal mutated base) was calculated following SantaLucia and Hicks (2004) and compared to both overall rate and transversion rate. There was no correlation in either comparison (*r*^2^ = 0.032 and 8 × 10^−5^, respectively) or between rate and the stability of the just the 5′ trinucleotide within the heptanucleotide (*r*^2^ = 0.0176). Nor is there a correlation between rate and the difference in free stability between the heptanucleotide with the original base and the heptanucleotide with the new base (*r*^2^ = 0.0667). Although ΔG_37_ values were used, the relationship between ΔG and T is linear so the lack of correlation is not temperature dependent. Overall, there is no evidence that the free energy of the local helix is a factor in the variation in mutation dynamics.

**Fig. 3.**
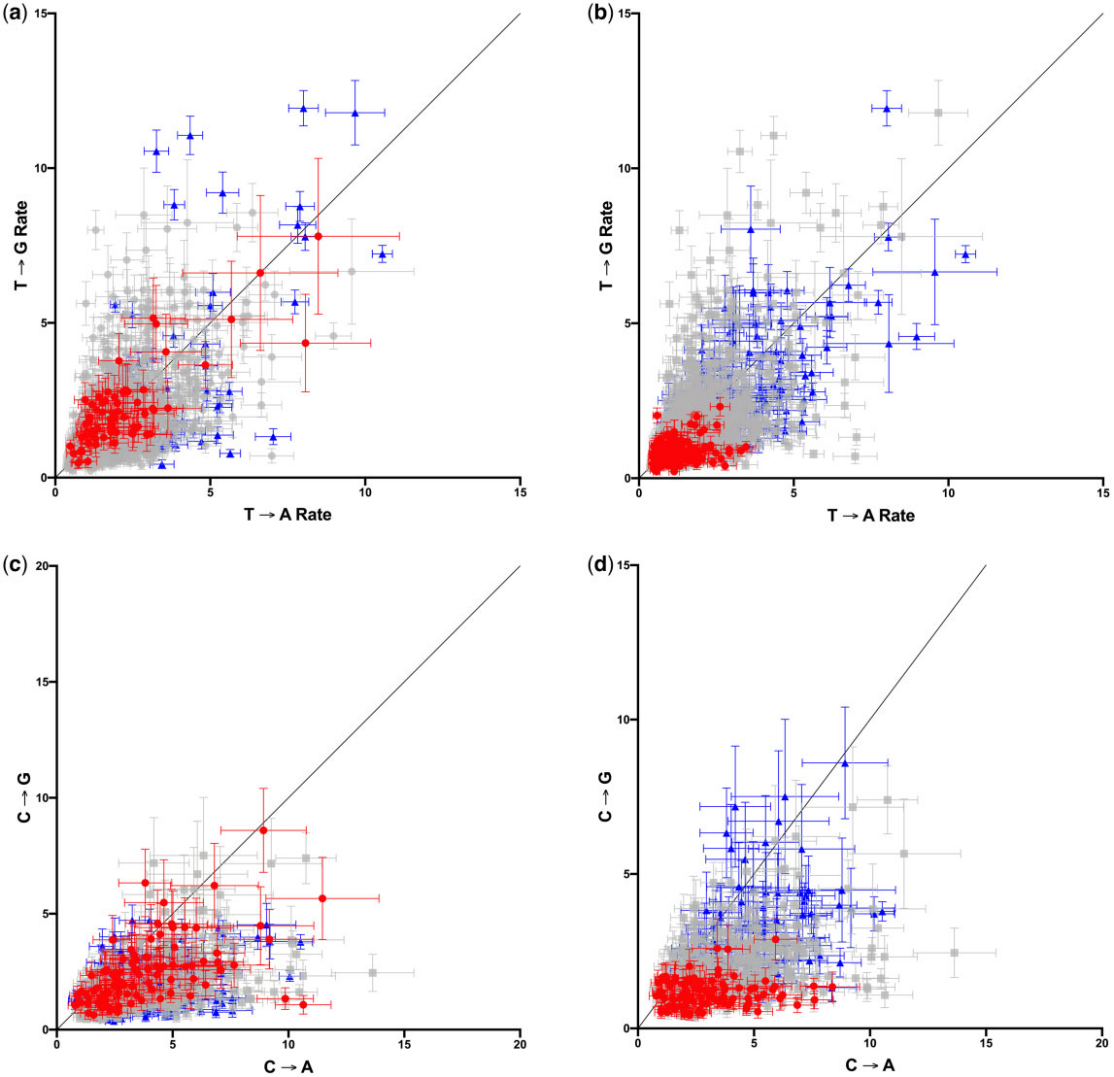
Substitution rate for the 2 transversions from T (a and b) and C (c and d) across hexanucleotide contexts in which there are at least 50 transversions. In (a) and (c), contexts with an of ATI ≤12 are in red (circles) and those with an ATI of 28 are in blue (triangles). These cutoff values were chosen simply to highlight contexts at the 2 ends of the A + T range. The separation of red and blue points demonstrates the effect of A + T content on rate. In (b) and (d), contexts with an RI of ≤6 are in red and those with an RI of ≥24 are in blue, also chosen to highlight contexts at the extremes of purine distribution. The separation of red and blue points demonstrates the effect of R distribution on rate. Equality lines are drawn in black. Rates tend to fall below this line for changes from C, indicating that C → A changes generally occur at higher than C → G changes.

For substitutions from both T and C, there is little correlation between the rates of transitions and transversions across contexts (*r*^2^ = 0.099 for T and *r*^2^ = 0.124 for C in Fig. 1, a and b) indicating that context does not influence mutation rate generally but, rather, affects transitions and transversions differently. However, a comparison of the 2 transversions from each of T and C shows no evidence that the 2 transversions vary differently across contexts (Fig. 3). The increased rate of transversion with increasing ATI and RI is observed for both T → G and T → A, and with increasing RI for both C → G and C → A, although C → A is predominantly greater than C → G. Thus, whatever context factors are affecting the rate of transversion do not appear to distinguish between transversion types.

One commonly observed effect of context on mutation is an increased rate of mutation at CpG sites as a result of a relatively rapid deamination of methylated Cs (Huttley 2004; Mugal *et al.* 2009; Francioli *et al.* 2015; Simon and Huttley 2020). There is some evidence for CG methylation in cpDNA (Muniandy *et al.* 2019), although how widespread this is remains unclear. In the 4,096 matrices, a roughly 37% increase in the rate of C → T substitution is observed on both strands in the CG context relative to the rate of C → T in other dinucleotide contexts (Table 2), which is significant at the 10^−6^ level using a χ^2^ test of heterogeneity. This is consistent with a CG effect in cpDNA, as would be expected if there is a significant level of methylation at CG sites, although the results are not direct evidence for methylation. It should be noted that the analysis was limited to intergenic regions over 70 nucleotides in length. If the frequency of CpG methylation within an intergenic region were associated with the length of that region, such as would be the case if sites closer to coding regions tend to be methylated at a higher frequency, then the measure of any CG effect could be biased.

**Table 2.** Comparison of the rate of C → T substitution in CpG contexts to the rate in other contexts.

	G → A	G → B^a^
CG	41,377	633,155
DG	184,227	3,835,559

	**OR = 1.36**	

	**C → T**	**C → V**

CG	41,607	633,520
CH	183,849	3,838,041

	**OR = 1.37**	

a D = A, G, or T (i.e. not C); B = not A; V = not T; H = not G.

The data suggest that there could be CpG methylation in noncoding cpDNA of flowering plants and that this contributes to some of the variation in substitutions across contexts that is observed in the data. However, the exclusion of those heptanucleotides with a potential substitution of a methylated cytosine does not change the general patterns in Figs. 1 and 2 and so the general context effects are not driven solely by any CG effect. Furthermore, although local A + T content, purine/pyrimidine arrangement, and a potential CG effect all contribute to the variation across contexts, the data in Figs. 1 and 2, in particular the wide range of Tv rate across contexts with an ATI of 28 (blue in Fig. 1, a and c), indicate that there must be additional factors that influence variation in substitutions.

### Context and composition equilibrium

**Fig. 4.**
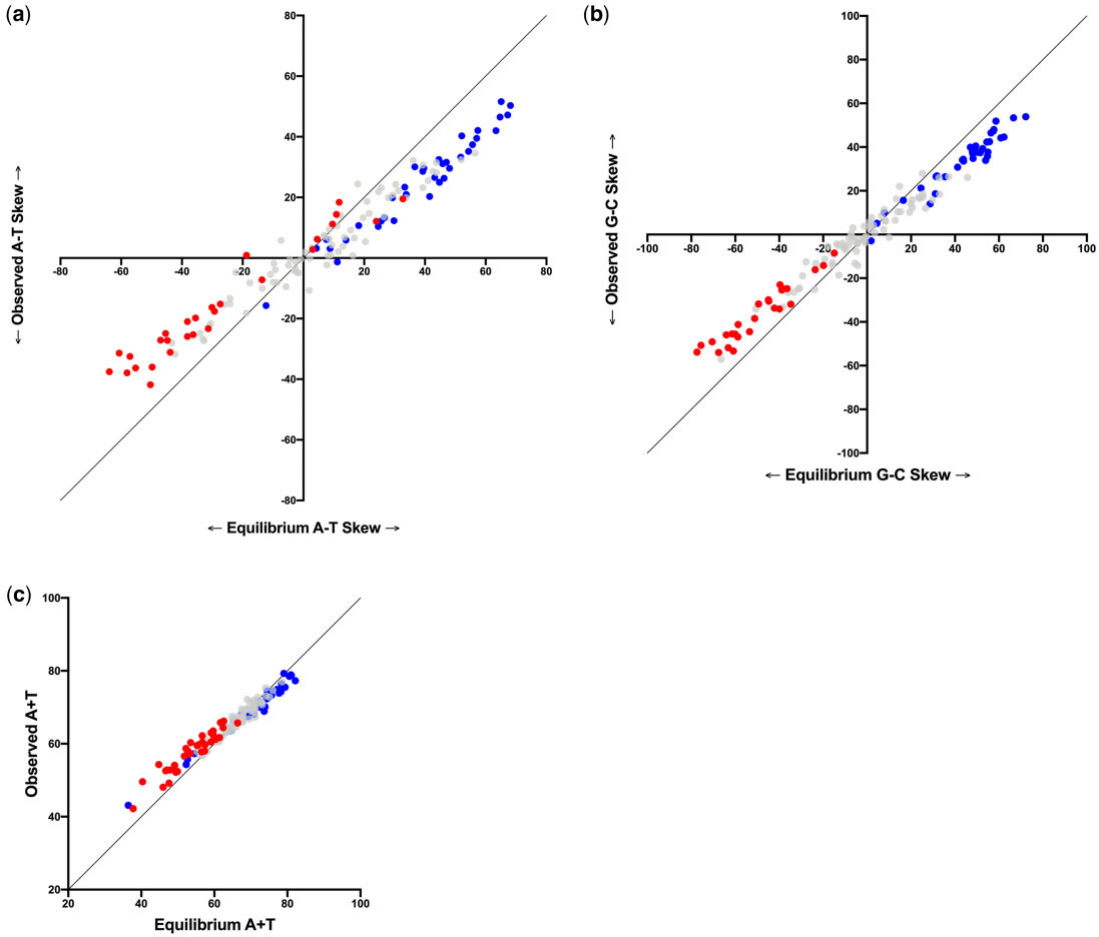
Stationary vector (predicted) and observed compositions across tetranucleotide contexts. Compositions are (a) A–T skew, (b) G–C skew, and (c) A + T content. The equality line for each plot is shown in black. To illustrate the relationship between flanking base content and evolutionary trajectory, contexts are colored depending on the composition of the 2 bases immediately flanking the substitution. In (a) and (b), the contexts with no purines immediately flanking the substitution are shown in red while those with 2 purines immediately flanking the substitution are shown in blue. In (c), the contexts with a + T = 0 content in the 2 bases immediately flanking the substitution are shaded red, while those with an A + T = 2 content in the 2 immediately flanking bases are shaded blue.

Along with analyses of rates, it is informative to compare substitution dynamics more generally across contexts by converting each substitution matrix to a transition matrix Π and then determining the stationary vector ϕ for which ϕ = ϕΠ (Morton 2003, 2022a). This represents the equilibrium base composition of sites evolving within that context, which provides information about the evolutionary trajectories of sites in different contexts as well as the degree to which we expect compositional variation at different neutral sites. A similar approach using simulation showed an excellent agreement between predicted and observed context-dependent composition in bacteria (Sung *et al.* 2015). In our study, complementary matrices are redundant and so only one of each pair was included in this analysis. A comparison of predicted equilibrium A + T context, G–C skew, and A–T skew to the observed composition in each hexanucleotide context is shown in Supplementary Fig. 3. However, many of these vectors have predicted base composition of 0% for one of more nucleotides because of low sample number in that context. Therefore, the same comparisons are shown in Fig. 4 for just the tetranucleotide contexts (i.e. excluding the information in the N_3_ neighboring pair), which have a larger sample size and also show the same general trends as the hexanucleotide contexts.

The data show several interesting features. First, there is a great deal of heterogeneity in the evolutionary trajectories of the substitution dynamics in different contexts: predicted equilibrium A + T content ranges from 36.4% to 82.8%, predicted G–C skew ranges from −77.4 to 72.2, and predicted A–T skew ranges from −63.9 to 68.2. Second, this variation is associated with the compositional features of the context. As seen in Fig. 4, the A + T content of the immediate neighboring pair (the N_1_ pair) is associated with the predicted equilibrium A + T content, and the R content on the same strand at the 2 immediately neighboring sites is associated with predicted skew, with sites surrounded by a purine on the same strand on each side expected to evolve to a stronger R-Y skew. The latter is consistent with the R[Y]R rate effect described above. Most intriguing, there is a very strong correlation, as well as a strong agreement, between the predicted and observed context-dependent composition across contexts, although for all 3 composition features analyzed, the agreement between predicted and observed shows more deviation in contexts at the extremes of the composition range.

The heterogeneity observed in Fig. 4 illustrates the importance of understanding context dependency of mutations and/or substitutions. In particular, measurements of selection that rely on a nucleotide substitution model could be significantly misled if they do not account for context (see also Sung *et al.* 2015). This was demonstrated recently for analyses of codon usage in chloroplast genes (Morton 2022a) and there could be much broader implications.

### Effect of the N_3_ and N_4_ neighboring pairs on substitutions

**Fig. 5.**
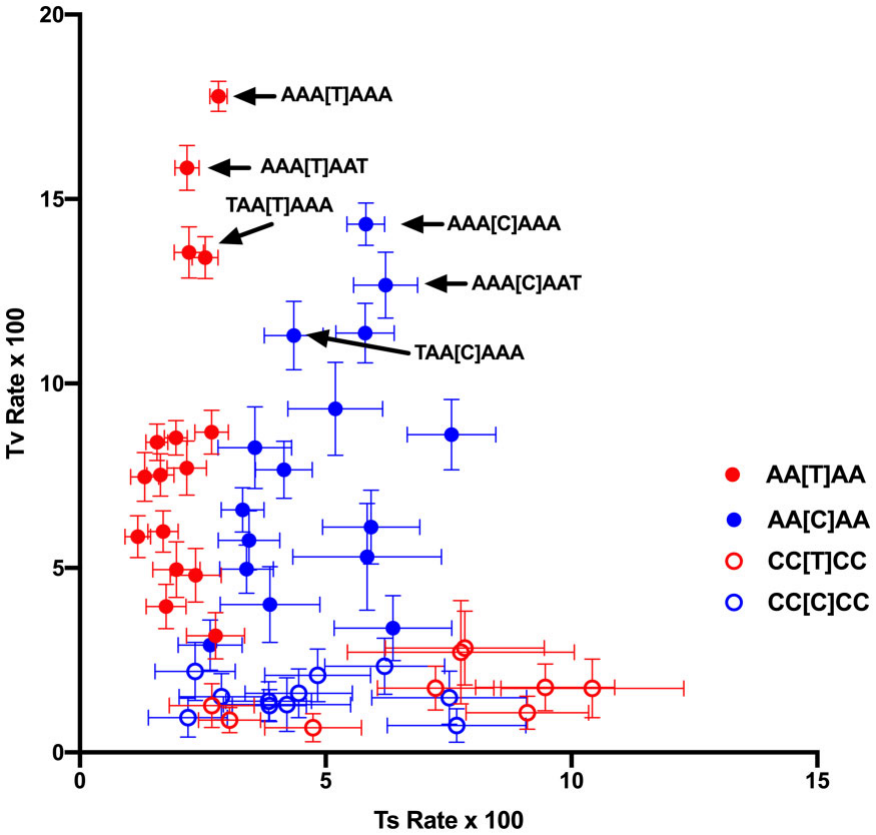
Transition and transversion rates in relation to the content of the N3 dinucleotide (the nucleotide pair 3 sites removed from the substitution as described in the text), for the internal tetranucleotides AA[Y]AA and CC[Y]CC. Substitutions from T (red) are differentiated from substitutions from C (blue). Closed circles represent the internal tetranucleotide AA[Y]AA and open circles represent the internal tetranucleotide CC[Y]CC. Some specific contexts are indicated.

**Fig. 6.**
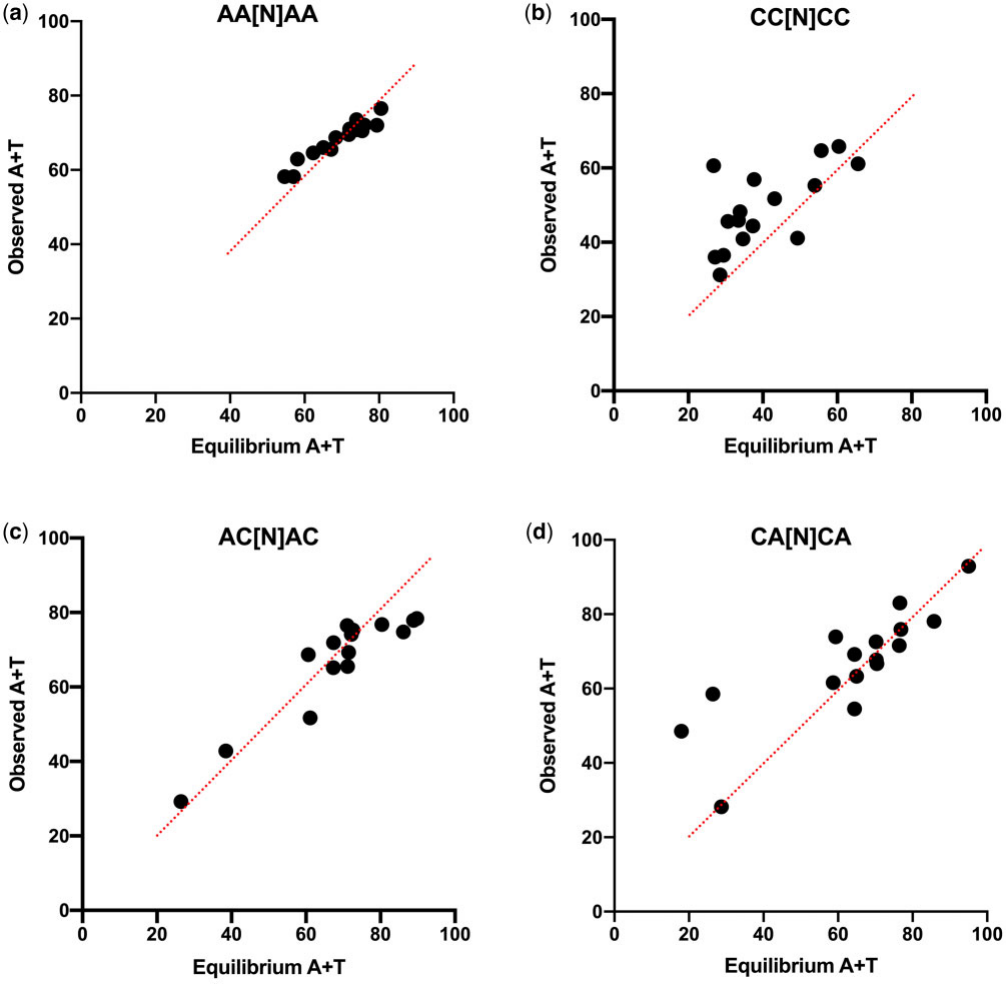
Predicted and observed A + T content across the 16 different N_3_ dinucleotides. Only contexts with at least 100 total substitutions are included. Each of the 4 plots controls for a different internal tetranucleotide context as indicated. Tetranucleotide contexts are shown as N_2_N_1_[N_0_]N_1_N_2_ where N_0_ indicates the substitution site. The N_3_ dinucleotide is composed of the 2 nucleotides immediately flanking this tetranucleotide, one 5′ and one 3′.

An association between substitutions and the neighboring bases up to with 4 nucleotides removed from the substitution site has been observed in other studies (Aggarwala and Voight 2016; Zhu *et al.* 2017; Simon and Huttley 2020; Morton 2022b). To study the direct influence of neighbors that are further removed requires that we control for the influence of the nucleotides that are closer to the substitution (Simon and Huttley 2020; Morton 2022b). As defined in the *Materials and* *Methods*, the N_**3**_ neighboring pair is composed of the 5′ and 3′ flanking nucleotides that are 3 bases removed from the substitution site. Internal to this pair is a pentanucleotide composed of the substitution site within a tetranucleotide context. The direct effect of the N_**3**_ pair on substitutions is examined here by comparing substitutions to the composition of the 16 N_3_ dinucleotides surrounding any specific pentanucleotide, which has the effect of controlling for the internal context. Rate variation across the 16 N_3_ contexts is shown in Fig. 5 for the internal pentanucleotides AA[T]AA, CC[T]CC, AA[C]AA, and CC[C]CC, which are at the extremes of the range of A + T and purine contents of the internal tetranucleotide context. In all 4 cases, there is significant variation across N_3_ contexts in both Ts and Tv rates. In general, the AA[N]AA context has a higher Tv rate than CC[N]CC, and AA[C]AA has a higher Ts rate than AA[T]AA. For the latter 2 pentanucleotides, the N_3_ pairs with the highest A + T content are associated with higher transversion rates. Figure 6 compares the equilibrium composition and the observed composition across N_3_ contexts for the internal contexts AA[N]AA, AC[N]AC, CA[N]CA, and CC[N]CC. In all cases, there is significant variation across N_3_ contexts in substitution dynamics as measured by equilibrium base composition and this variation agrees well with the observed composition.

**Fig. 7.**
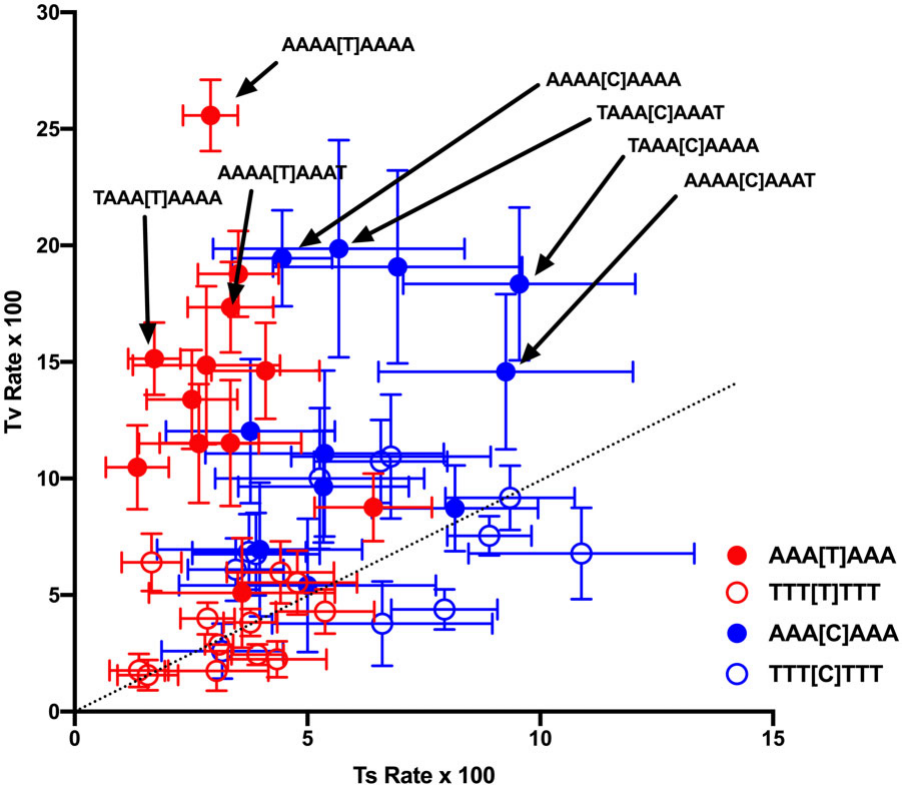
Transition and transversion rates across different N_4_ dinucleotides. Two internal hexanucleotides are controlled for and substitutions from T (red) are differentiated from substitutions from C (blue). Similar to Fig. 6, the closed circles represent the internal hexanucleotide AAA[N]AAA and the open circles represent the internal hexanucleotide TTT[N]TTT. The N_4_ dinucleotide is composed of the 2 nucleotides immediately flanking this hexanucleotide, one 5′ and one 3′. Some specific contexts are indicated.

A similar analysis of the effect of what we can call the N_4_ pair shows that these 2 nucleotides also directly influence substitutions. Using the same approach described for heptanucleotides, substitution matrices were generated by treating sites as the centers of nonanucleotides, providing data on the octonucleotide context. Controlling for the internal hexanucleotide context limits the number of matrices that have a large enough sample size but there were several, particularly for A + T-rich internal hexanucleotide contexts, that could be utilized. The data for the 16 N_4_ dinucleotides surrounding each of the AAA[Y]AAA and TTT[Y]TTT heptanucleotides show that the composition of the N_4_ pair is associated with rate variation (Fig. 7). Most noticeably, higher rates of transversion from the sequences AAA[T]AAA and AAA[C]AAA are associated with higher A + T content of the N_4_ pair. Although there are not enough data to give us information about an influence of the N_4_ pair across all internal contexts, the data indicate that there is some level of influence of these 2 nucleotides on substitutions.

Figures 6 and 7 show that both the N_3_ and N_4_ pairs have a significant influence on substitutions beyond any influence of, or compositional correlation with, the nucleotides closer to the substitution site. The data discussed above showed no evidence for a relationship between local helix stability, as measured by the method of SantaLucia and Hicks (2004) that is based on the ΔG contributions of dinucleotides in dsDNA molecule. Thus, it does not seem to be the case that the N_3_ and N_4_ pairs have an effect through an influence on stability, although it is possible that these ΔG values do not accurately measure stability of the new dsDNA molecule during replication at the site of incorporation or during DNA repair. One possibility is that the N_3_ and N_4_ pairs have an influence on repair, such as mismatch repair, via sequence/structural features that interact with repair enzymes. Overall, however, it is not clear how the composition of these nucleotides affects substitution dynamics.

## Conclusions

Substitutions within intergenic regions display a complex relationship with the octonucleotide context in which they occur. The data demonstrate that the A + T content, the arrangement of purines vs pyrimidines along 1 strand of the octonucleotide, and the existence of a CpG at the substitution site, which could be the result of, are all associated with variation in substitution rate and bias. The context-dependent variation in substitution patterns is likely to be the result of a context dependency of the underlying mutations. Although there are probably selective constraints on some intergenic sites, it is expected that the vast majority of substitutions are the result of random fixation. Most importantly, selective constraints on some sites cannot generate the observed context dependency at those sites that vary, particularly the relationship between context and transition:transversion bias and between context and equilibrium composition.

This variation results in very different evolutionary trajectories at different sites, such that there is a wide range in predicted equilibrium composition across contexts. If we accept that these dynamics reflect the context variation of underlying mutations, then the composition of sites will vary dramatically in the absence of selection. This has profound implications for a number of analyses, particularly methods used to assess selection. As demonstrated recently, the study of selective pressure on codon usage, which is based on an expected neutral codon usage, requires that the context dependency of mutation be taken into account (Morton 2022a). Phylogenetic reconstruction and analyses of rate variation among lineages are also potentially biased if they fail to account for an influence of context.

Why and how the influence of context differs in different genomes remains unclear, but the evidence here that local helix stability shows no correlation with variation in substitution rate suggests that there may not be universal features of how context affects substitution bias. The influence of context observed here shows some similarity to what has been observed in other genomes (Huttley 2004; Sung *et al.* 2015; Aggarwala and Voight 2016; Simon and Huttley 2020), but it has also the case that context dependency varies across plastid lineages (Morton 2022b). It is possible that differences in polymerase misincorporation and/or proofreading, as well as differences in repair systems all contribute to context effects, and that these evolve over time. Although the full set of relationships between context and mutation remains somewhat uncertain, this analysis of context dependency demonstrates the importance of understanding how mutation rates vary across sites and how this variation affects sequence analyses that require a mutation model. The matrices generated here also provide a context-dependent model that can be applied in further studies of selection in cpDNA or other analyses that utilize a substitution model.

## Data availability

Alignment data are available at https://doi.org/10.5061/dryad.fj6q573x5.

Supplemental material is available at *G3* online.

## Supplementary Material

jkac150_Supplementary_Material_Legends

jkac150_Supplementary_Table_S1

jkac150_Supplementary_Figure_S1

jkac150_Supplementary_Figure_S2

jkac150_Supplementary_Figure_S3

## References

[jkac150-B1] Aggarwala V , Voight BF. An expanded sequence context model broadly explains variability in polymorphism levels across the human genome. Nat Genet. 2016;48(4):349–355. doi:10.1038/ng.3511. 26878723 PMC4811712

[jkac150-B2] Arndt PF , Burge CB, Hwa T. DNA sequence evolution with neighbor-dependent mutation. J Comput Biol. 2003;10(3–4):313–322. doi:10.1089/10665270360688039. 12935330

[jkac150-B3] Burgers PMJ , Kunkel TA. Eukaryotic DNA replication fork. Annu Rev Biochem. 2017;86:417–438. doi:10.1146/annurev-biochem. 28301743 PMC5597965

[jkac150-B4] Clegg MT. Chloroplast gene sequences and the study of plant evolution. Proc Natl Acad Sci USA. 1993;90(2):363–367. doi:10.1073/pnas.90.2.363. 8421667 PMC45662

[jkac150-B5] Cock PJA , Antao T, Chang JT, Chapman BA, Cox CJ, Dalke A, Friedberg I, Hamelryck T, Kauff F, Wilczynski B, et al Biopython: freely available Python tools for computational molecular biology and bioinformatics. Bioinformatics. 2009;25(11):1422–1423. doi:10.1093/bioinformatics/btp163. 19304878 PMC2682512

[jkac150-B6] Coulondre C , Miller JH, Farabaugh PJ, Gilbert W. Molecular basis of base substitution hotspots in Escherichia coli. Nature. 1978;274(5673):775–780. doi:10.1038/274775a0. 355893

[jkac150-B7] Cox DR , Miller HD. The Theory of Stochastic Processes. New York: Chapman and Hall/CRC; 2017.

[jkac150-B8] Ehrlich M , Wang RYH. 5-Methylcytosine in eukaryotic DNA. Science. 1981;212(4501):1350–1357. doi:10.1126/science.6262918. 6262918

[jkac150-B9] Francioli LC , Polak PP, Koren A, Menelaou A, Chun S, Renkens I, Van Duijn CM, Swertz M, Wijmenga C, Van Ommen G, et al; Genome of the Netherlands Consortium. Genome-wide patterns and properties of de novo mutations in humans. Nat Genet. 2015;47(7):822–826. doi:10.1038/ng.3292. 25985141 PMC4485564

[jkac150-B10] Ghosh S , Takahashi S, Endoh T, Tateishi-Karimata H, Hazra S, Sugimoto N. Validation of the nearest-neighbor model for Watson-Crick self-complementary DNA duplexes in molecular crowding condition. Nucleic Acids Res. 2019;47(7):3284–3294. doi:10.1093/nar/gkz071. 30753582 PMC6468326

[jkac150-B11] Huttley GA. Modeling the impact of DNA methylation on a the evolution of BRCA1 in mammals. Mol Biol Evol. 2004. doi:10.1093/molbev/msh187. 15190129

[jkac150-B12] Kunkel TA , Erie DA. Eukaryotic mismatch repair in relation to DNA replication. Annu Rev Genet. 2015;49:291–313. doi:10.1146/annurev-genet-112414-054722. 26436461 PMC5439269

[jkac150-B13] Lobry JR , Louarn JM. Polarisation of prokaryotic chromosomes. Curr Opin Microbiol. 2003;6(2):101–108. doi:10.1016/S1369-5274(03)00024-9. 12732297

[jkac150-B14] Mendelman LV , Boosalis MS, Petruska J, Goodman MF. Nearest neighbor influences on DNA polymerase insertion fidelity. J Biol Chem. 1989;264(24):14415–14423. doi:10.1016/S0021-9258(18)71695-5. 2474545

[jkac150-B15] Michaelson JJ , Shi Y, Gujral M, Zheng H, Malhotra D, Jin X, Jian M, Liu G, Greer D, Bhandari A, et al Whole-genome sequencing in autism identifies hot spots for de novo germline mutation. Cell. 2012;151(7):1431–1442. doi:10.1016/j.cell.2012.11.019. 23260136 PMC3712641

[jkac150-B16] Morley SA , Ahmad N, Nielsen BL. Plant organelle genome replication. Plants. 2019;8(10):358. doi:10.3390/plants8100358. PMC6843274 31546578

[jkac150-B17] Morton BR. Neighboring base composition and transversion/transition bias in a comparison of rice and maize chloroplast noncoding regions. Proc Natl Acad Sci USA. 1995;92(21):9717–9721. doi:10.1073/pnas.92.21.9717. 7568204 PMC40873

[jkac150-B18] Morton BR. The role of context-dependent mutations in generating compositional and codon usage bias in grass chloroplast DNA. J Mol Evol. 2003;56(5):616–629. doi:10.1007/s00239-002-2430-1. 12698298

[jkac150-B19] Morton BR. Context-dependent mutation dynamics, not selection, explains the codon usage bias of most angiosperm chloroplast genes. J Mol Evol. 2022a;90(1):17–29. doi:10.1007/s00239-021-10038-w. 34932159 PMC8821512

[jkac150-B20] Morton BR. Context-dependent substitution dynamics in plastid DNA across a wide range of taxonomic groups. J Mol Evol. 2022b;90(1):44–55. doi:10.1007/s00239-021-10040-2. 35037071

[jkac150-B21] Morton BR , Bi IV, McMullen MD, Gaut BS. Variation in mutation dynamics across the maize genome as a function of regional and flanking base composition. Genetics. 2006;172(1):569–577. doi:10.1534/genetics.105.049916. 16219784 PMC1456184

[jkac150-B22] Morton BR , Clegg MT. Neighboring base composition is strongly correlated with base substitution bias in a region of the chloroplast genome. J Mol Evol. 1995;41(5):597–603. doi:10.1007/BF00175818. 7490774

[jkac150-B23] Morton RA , Morton BR. Separating the effects of mutation and selection in producing DNA skew in bacterial chromosomes. BMC Genomics. 2007;8:369. doi:10.1186/1471-2164-8-369. 17935620 PMC2099444

[jkac150-B24] Mugal CF , Ellegren H. Substitution rate variation at human CpG sites correlates with non-CpG divergence, methylation level and GC content. Genome Biol. 2011;12(6):R58. doi:10.1186/gb-2011-12-6-r58. 21696599 PMC3218846

[jkac150-B25] Mugal CF , Von Grünberg HH, Peifer M. Transcription-induced mutational strand bias and its effect on substitution rates in human genes. Mol Biol Evol. 2009;26(1):131–142. doi:10.1093/molbev/msn245. 18974087

[jkac150-B26] Muniandy K , Tan MH, Song BK, Ayub Q, Rahman S. Comparative sequence and methylation analysis of chloroplast and amyloplast genomes from rice. Plant Mol Biol. 2019;100(1–2):33–46. doi:107/s11103-019-00841-x. 30788769 10.1007/s11103-019-00841-x

[jkac150-B27] Ossowski S , Schneeberger K, Lucas-Lledó JI, Warthmann N, Clark RM, Shaw RG, Weigel D, Lynch M. The rate and molecular spectrum of spontaneous mutations in Arabidopsis thaliana. Science. 2010;327(5961):92–94. doi:10.1126/science.1180677. 20044577 PMC3878865

[jkac150-B28] SantaLucia J , Hicks D. The thermodynamics of DNA structural motifs. Annu Rev Biophys Biomol Struct. 2004. doi:10.1146/annurev.biophys.32.110601.141800. 15139820

[jkac150-B29] Simon H , Huttley G. Quantifying influences on intragenomic mutation rate. G3 (Bethesda). 2020;10(8):2641–2652. doi:10.1534/g3.120.401335. PMC7407452 32527747

[jkac150-B30] Smith TCA , Arndt PF, Eyre-Walker A. Large scale variation in the rate of germ-line de novo mutation, base composition, divergence and diversity in humans. PLoS Genet. 2018;14(3):e1007254. doi:10.1371/journal.pgen.1007254. 29590096 PMC5891062

[jkac150-B31] Sung W , Ackerman MS, Gout JF, Miller SF, Williams E, Foster PL, Lynch M. Asymmetric context-dependent mutation patterns revealed through mutation-accumulation experiments. Mol Biol Evol. 2015;32(7):1672–1683. doi:10.1093/molbev/msv055. 25750180 PMC4476155

[jkac150-B32] Zacharias M. Base-pairing and base-stacking contributions to double-stranded DNA formation. J Phys Chem B. 2020;124(46):10345–10352. doi:10.1021/acs.jpcb.0c07670. 33156627

[jkac150-B33] Zheng T , Ichiba T, Morton BR. Assessing substitution variation across sites in grass chloroplast DNA. J Mol Evol. 2007;64(6):605–613. doi:10.1007/s00239-006-0076-0. 17541677

[jkac150-B34] Zhu Y , Neeman T, Yap VB, Huttley GA. Statistical methods for identifying sequence motifs affecting point mutations. Genetics. 2017;205(2):843–856. doi:10.1534/genetics.116.195677. 27974498 PMC5289855

