# Supplementary material for: Substitution rate heterogeneity across hexanucleotide contexts in noncoding chloroplast DNA
Source: G3 (Bethesda). 2022 Jun 14;12(8):jkac150. doi: 10.1093/g3journal/jkac150 (PMC9339276; doi:10.1093/g3journal/jkac150)
Supplement: jkac150_Supplementary_Material_Legends [file jkac150_supplementary_material_legends.docx]

**Supplementary Material**

**Table S1:** Contexts with 25 highest and 25 lowest rates of substitution.

**Figure S1:** Rates of substitution with 95% CI from T and C across hexanucleotide contexts with at least 50 transitions and 50 transversions

**Figure S2:** Ts:Tv for substitutions of T and C relative to the ATI, RI, and the RATI values of the hexanucleotide context.

**Figure S3:** Predicted equilibrium and observed composition within each hexanucleotide context.
