# Supplementary material for: Substitution rate heterogeneity across hexanucleotide contexts in noncoding chloroplast DNA
Source: G3 (Bethesda). 2022 Jun 14;12(8):jkac150. doi: 10.1093/g3journal/jkac150 (PMC9339276; doi:10.1093/g3journal/jkac150)
Supplement: jkac150_Supplementary_Table_S1 [file jkac150_supplementary_table_s1.pdf]

Table S1

a) Contexts with the highest 25 rates of transitions, transversions and total substitution of Ts

Context[1]	Ts Rate x 100	Context	Tv Rate x 100	Context	Rate x 100
ACC[T]GAA	15.6	TTT[T]AAA	21.5	TTT[T]AAA	29.6
ACC[T]TTT	10.6	ATA[T]AAA	19.9	ATT[T]AAA	24.9
CCC[T]TTT	9.3	AAA[T]AAA	17.8	ATA[T]AAA	24.0
ATT[T]AAA	9.0	AAT[T]AAA	16.7	ACC[T]GAA	23.5
AAC[T]GAT	8.5	ATT[T]AAA	16.0	AAT[T]AAA	22.7
ATC[T]GAA	8.5	AAA[T]AAT	15.8	TTT[T]AAT	20.9
ACC[T]GAT	8.2	TTT[T]AAT	15.4	AAA[T]AAA	20.6
AGA[T]GGT	8.2	ATG[T]AAT	14.9	TTG[T]AAT	19.9
TTT[T]AAA	8.1	TTA[T]AAA	14.6	ATG[T]AAT	18.1
ATT[T]TAA	8.0	CTA[T]AAA	13.9	AAA[T]AAT	18.0
GCC[T]TTT	7.9	TTA[T]AAT	13.8	TTA[T]AAA	17.6
TGG[T]GGA	7.8	AAA[T]AAG	13.6	AAC[T]GAT	16.8
GTT[T]AAA	7.6	TAA[T]AAA	13.4	GTT[T]AAA	16.8
ATG[T]TTT	7.6	AGA[T]AAA	13.0	ACT[T]AAA	16.2
ACA[T]GAT	7.6	TTG[T]AAT	13.0	TGT[T]AAA	16.2
ATC[T]GGA	7.5	ATA[T]AAT	12.7	TAA[T]AAA	16.0
ATC[T]GTT	7.1	AAG[T]AAA	11.4	CTA[T]AAA	15.8
AGG[T]CTT	7.1	TAG[T]AAT	11.3	AGT[T]AAA	15.8
ATA[T]CCT	7.0	ATT[T]AAT	11.1	AAA[T]AAG	15.8
TTG[T]AAT	6.9	AGT[T]AAA	11.1	ATT[T]AAT	15.7
ATC[T]GTA	6.8	ACT[T]AAA	10.9	ATA[T]AAT	15.7
ATC[T]ACA	6.7	TGA[T]AAA	10.8	TTA[T]AAT	15.7
GGA[T]AGT	6.6	TGT[T]AAA	10.6	AGA[T]AAA	15.0
TTC[T]CCA	6.6	AAT[T]AAT	10.6	ACC[T]GAT	14.9
AAC[T]TTT	6.4	ACA[T]AAA	10.3	TTC[T]AAA	14.2
N1 R = 0.96[2]<br>N1 A+T = 0.80[3]		N1 R = 1.64<br>N1 A+T = 1.84		N1 R = 1.48<br>N1 A+T = 1.64	

1 – Contexts are shown as a heptanucleotide with the substituted T (bracketed) at the center
2 – Average Purine content of the two nucleotides immediately flanking the substitution site
3 – Average A+T content of the two nucleotides immediately flanking the substitution site

b) Contexts with the lowest 25 rates of transitions, transversions and total substitution of Ts

Context	Ts Rate x 100	Context	Tv Rate x 100	Context	Rate x 100
TCT[T]TCA	0.64	TAT[T]CCA	0.66	TCT[T]TCA	1.41
CCT[T]TTC	0.73	AAT[T]CGA	0.72	TCT[T]TCT	1.70
GAA[T]TGA	0.74	TCT[T]TCA	0.77	CCT[T]TTC	1.75
TTT[T]TGG	0.80	TCT[T]TCT	0.79	TAT[T]CCA	1.78
TCA[T]ATA	0.81	GAT[T]CAT	0.81	TTA[T]TCC	1.82
TAA[T]TCA	0.84	GAT[T]CGA	0.81	GAT[T]TTC	1.95
GAT[T]TCT	0.86	TTA[T]TCC	0.85	GAT[T]TCT	1.99
CAA[T]AGA	0.88	CTC[T]TTC	0.90	CTC[T]TTC	2.01
TCA[T]TAT	0.89	ATC[T]CTT	0.90	AGA[T]CTT	2.05
TTA[T]TGT	0.89	GAT[T]TTC	0.92	GAA[T]TCT	2.06
GAA[T]AGG	0.90	AAT[T]CTC	0.92	GAA[T]CAA	2.07
GAA[T]TGG	0.90	AGA[T]CTT	0.92	GAA[T]TGG	2.07
TAA[T]ATT	0.91	AAT[T]CTT	0.93	GTA[T]TTC	2.08
TCT[T]TCT	0.92	GAT[T]CTA	0.99	GAA[T]TGA	2.16
AAA[T]TGA	0.92	GAA[T]CAA	1.00	TCT[T]CTT	2.20
TTA[T]TGA	0.93	AAT[T]TCA	1.01	CCA[T]TTC	2.28
GAA[T]ATT	0.95	CCT[T]CTT	1.01	TCT[T]TGT	2.28
CTT[T]TTG	0.95	TAT[T]CTT	1.02	GAT[T]CGA	2.29
TTA[T]TCC	0.97	CCT[T]TTC	1.02	CTA[T]TTC	2.34
TCT[T]TGT	0.97	TAT[T]CCT	1.04	AAT[T]CGA	2.39
GTA[T]TTC	0.98	ATT[T]CGA	1.06	CTT[T]TTG	2.39
GAA[T]TCT	0.99	CTT[T]CTT	1.06	TAA[T]TGT	2.41
CAA[T]ATT	1.00	GAA[T]TCT	1.07	GGA[T]TTC	2.44
CAA[T]TAT	1.00	CTT[T]TCA	1.08	CAT[T]TCT	2.45
TAA[T]TCT	1.00	CCT[T]TCT	1.09	CAA[T]TGA	2.46
N1 R = 0.96<br>N1 A+T = 2.0		N1 R = 0.16<br>N1 A+T = 1.32		N1 R = 0.48<br>N1 A+T = 1.72	

c) Contexts with the highest 25 rates of transitions, transversions and total substitution of Cs

Context	Ts Rate x 100	Context	Tv Rate x 100	Context	Rate x 100
AAA[C]GTT	20.1	ATG[C]ATT	18.2	AAA[C]GGG	35.4
AAA[C]GGG	17.8	AAA[C]GGG	17.5	TAG[C]GTA	33.0
AAA[C]GCA	17.2	TAG[C]GTA	16.4	AAA[C]GTT	32.0
TAG[C]GTA	16.7	AAA[C]TGA	16.1	TAT[C]GGT	28.2
TAT[C]GGT	14.8	AAA[C]AAA	14.3	TAA[C]GAT	25.3
AAA[C]TGT	14.3	AAA[C]TGG	14.0	AAA[C]AGC	24.2
ACT[C]TCT	13.9	AAG[C]AAA	13.9	AAA[C]TGG	23.8
TAA[C]GAT	13.7	TTG[C]AAA	13.6	AAA[C]GCA	23.6
ATA[C]GGA	13.1	AAA[C]TAG	13.6	AAA[C]TGA	23.5
TTA[C]CTT	12.6	TAT[C]GGT	13.4	TTG[C]GAT	23.3
AAA[C]GAG	12.4	AGT[C]TAG	13.4	AAA[C]GAG	23.2
TTT[C]GGT	12.3	AAA[C]ACC	13.0	TAA[C]AGA	22.9
TTT[C]GGC	12.2	AAA[C]ATT	13.0	TTT[C]GGC	22.7
ATA[C]GTA	12.0	AGA[C]TAA	12.9	AAA[C]TGT	22.1
TAA[C]GGA	11.8	AAA[C]AGC	12.8	AAA[C]ATT	22.0
TTG[C]GAT	11.6	AAT[C]GAA	12.7	AAA[C]ACC	21.8
CCC[C]TTT	11.6	TAA[C]AAA	12.7	ATG[C]GAA	21.6
AAA[C]GTA	11.5	AAT[C]AAA	12.4	ATG[C]ATT	21.2
AAA[C]AGC	11.5	TTC[C]AAA	12.1	AAA[C]GTA	21.1
TAT[C]AGT	11.4	AAA[C]GTT	11.8	ACG[C]GAA	20.9
ATG[C]GAA	11.2	TAA[C]AGA	11.8	TAA[C]AGT	20.8
CAA[C]GAT	11.1	TCC[C]AAA	11.7	AAA[C]GAA	20.7
TAA[C]CTA	11.1	TTG[C]GAT	11.6	AAA[C]AAA	20.1
TAA[C]AGA	11.1	AGT[C]TAA	11.6	TAT[C]AGT	20.1
AGG[C]GAA	11.0	AAA[C]AGG	11.5	AGT[C]TAG	20.0
N1 R = 1.56<br>N1 A+T = 1.04		N1 R = 1.48<br>N1 A+T = 1.48		N1 R = 1.76<br>N1 A+T = 1.28	

d) Contexts with the lowest 25 rates of transitions, transversions and total substitution of Cs

Context	Ts Rate x 100	Context	Tv Rate x 100	Context	Rate x 100
GAT[C]TAA	1.30	ATC[C]ATG	1.14	GAT[C]TAA	3.48
GTT[C]AAT	1.41	ATC[C]CTT	1.17	GTT[C]ATA	3.73
CCT[C]TTA	1.71	GAT[C]CAT	1.26	GAT[C]CAT	3.77
GTT[C]ATA	1.72	GAA[C]TTA	1.28	TTC[C]ATG	3.84
TCT[C]AAA	1.79	TTC[C]CTT	1.39	AAC[C]CAA	3.95
ATT[C]ATG	1.79	CCC[C]TTT	1.47	TAT[C]AAC	4.20
GGT[C]AAA	1.85	AAC[C]CAA	1.47	ATC[C]ATG	4.28
GAT[C]AAA	1.91	TAT[C]CTT	1.53	CAT[C]CAA	4.31
ATT[C]AAC	1.98	GAT[C]TTC	1.56	GAA[C]TTA	4.35
GAT[C]TAT	1.99	GAT[C]CTT	1.60	TTC[C]CTC	4.43
TAT[C]AAC	2.06	TCC[C]CTT	1.76	GAT[C]TTC	4.53
TTC[C]ATG	2.06	ATT[C]CAT	1.78	ATT[C]CAG	4.53
ACT[C]GAT	2.06	TTC[C]ATG	1.79	TAC[C]TAA	4.58
AGT[C]AAA	2.07	TTC[C]CAT	1.85	CCT[C]TTA	4.58
CTA[C]TAA	2.10	ATT[C]CAG	1.85	ATT[C]ATG	4.58
AGC[C]AAA	2.10	CCT[C]CTT	1.85	TCC[C]CTT	4.65
TAT[C]ACA	2.13	TTA[C]CTA	1.87	TCT[C]ATA	4.72
TGT[C]ATA	2.19	TTC[C]ACA	1.88	ATT[C]TGT	4.73
GAT[C]GAG	2.20	TAC[C]TAA	1.90	TCT[C]ATC	4.73
CTA[C]AAT	2.20	GAT[C]TTG	1.93	ATC[C]TAG	4.79
TCT[C]ATA	2.22	CAA[C]TTT	1.95	ATT[C]CAT	4.79
CAT[C]CAA	2.23	TTC[C]CTC	1.97	GAT[C]GAG	4.80
AGT[C]AAG	2.24	TAT[C]CCT	2.01	GAT[C]TAT	4.82
CTT[C]TAC	2.24	GTT[C]ATA	2.01	GTT[C]AAT	4.86
CAT[C]AAA	2.26	TTC[C]TTC	2.02	GAT[C]TTG	4.88
N1 R = 0.84<br>N1 A+T = 1.80		N1 R = 0.28<br>N1 A+T = 1.00		N1 R = 0.36<br>N1 A+T = 1.40	

e) Contexts with the highest 25 Ts:Tv values from T and C

Context	Ts:Tv	Context	Tv:Tv
ATC[T]GTT	4.73	CCC[C]TTT	7.90
ATC[T]CTT	4.08	ATC[C]CTT	4.44
ACC[T]TTT	4.04	ACC[C]TTT	3.98
GCC[T]TTT	3.75	TTC[C]CTT	3.90
CCC[T]TTT	3.71	TAT[C]CTT	3.64
CCC[T]TTC	3.57	CTC[C]TTT	3.51
AAC[T]TTT	3.45	ATC[C]TTC	3.34
ATA[T]CCT	3.20	TTC[C]TTC	3.34
TAT[T]CCT	3.02	CTA[C]TTT	3.31
ATC[T]GTA	2.96	ATC[C]GTT	3.21
TTC[T]CCA	2.92	ATC[C]GTA	3.16
AAT[T]CTT	2.83	TTA[C]CTT	3.14
ATC[T]ACA	2.79	TCC[C]TTT	3.03
CAA[T]CCA	2.65	ATC[C]TTT	2.98
ATC[T]ATG	2.55	CAT[C]TTT	2.81
AAA[T]CCC	2.43	GAA[C]TTT	2.81
ATC[T]TTT	2.33	CTT[C]TCT	2.76
AAT[T]CGA	2.30	ATC[C]ACA	2.75
TAT[T]CTT	2.27	ATC[C]ATG	2.75
ATC[T]CTA	2.25	CAA[C]TTT	2.74
AAT[T]CCT	2.25	TTA[C]TCT	2.73
ATC[T]GGA	2.23	AAA[C]GCA	2.71
GAT[T]CAT	2.23	TAC[C]ATA	2.71
ATT[T]GAC	2.20	TTT[C]GAC	2.70
TTG[T]TCT	2.18	ATA[C]CTT	2.64
N1 A+T =0.76		N1 A+T = 1.04	
N1 R = 0.40		N1 R = 0.56	

e) Contexts with the lowest 25 Ts:Tv values from T and C

Context	Ts:Tv	Context	Tv:Tv
CTA[T]AAA	0.13	ATG[C]ATT	0.17
AAA[T]AAT	0.14	AGT[C]AAA	0.19
TTA[T]AAT	0.14	AAT[C]AAA	0.23
AGA[T]AAA	0.15	AGT[C]TAA	0.24
TAG[T]AAT	0.15	ATG[C]AAA	0.26
TCA[T]AAT	0.15	ATG[C]AAT	0.26
AAA[T]AAA	0.16	TCT[C]AAA	0.28
AAA[T]AAG	0.16	ACT[C]AAA	0.29
TGA[T]AAA	0.16	AGA[C]TAA	0.30
AAA[T]AGG	0.17	TGA[C]AAA	0.30
AAA[T]ATG	0.17	AGA[C]TAT	0.31
TAA[T]ATT	0.17	ACT[C]TAA	0.32
AAA[T]AAC	0.18	ATA[C]AAA	0.32
TAG[T]ATA	0.18	TCC[C]AAA	0.32
AAA[T]TAG	0.19	ACA[C]AAA	0.33
AAG[T]AAG	0.19	ATT[C]AAA	0.33
TAA[T]AAA	0.19	AGT[C]AAG	0.33
TTA[T]AGA	0.19	CAT[C]AAA	0.33
TAA[T]AAT	0.19	TTC[C]AAA	0.34
ATA[T]AAA	0.20	AGT[C]AAT	0.35
AAA[T]ATC	0.20	CTT[C]AAA	0.35
AAA[T]ATT	0.20	AAA[C]TAG	0.36
TAA[T]AAG	0.20	AAG[C]AAA	0.37
TGA[T]AAT	0.20	AGA[C]AAA	0.37
CTA[T]ATT	0.21	AAG[C]AAT	0.37
N1 A+T =1.88		N1 A+T = 1.72	
N1 R = 1.96		N1 R = 1.24	
