# Supplementary material for: Substitution rate heterogeneity across hexanucleotide contexts in noncoding chloroplast DNA
Source: G3 (Bethesda). 2022 Jun 14;12(8):jkac150. doi: 10.1093/g3journal/jkac150 (PMC9339276; doi:10.1093/g3journal/jkac150)
Supplement: jkac150_Supplementary_Figure_S1 [file jkac150_supplementary_figure_s1.pdf]

Figure S1

a)

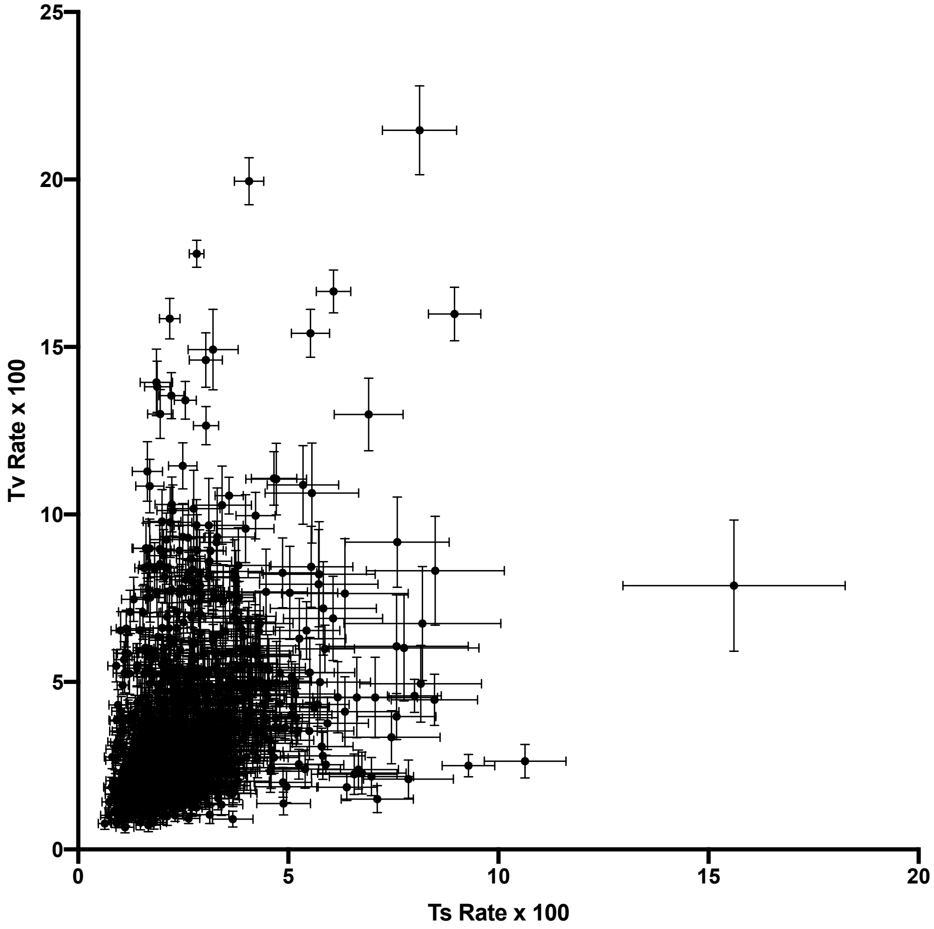

b)

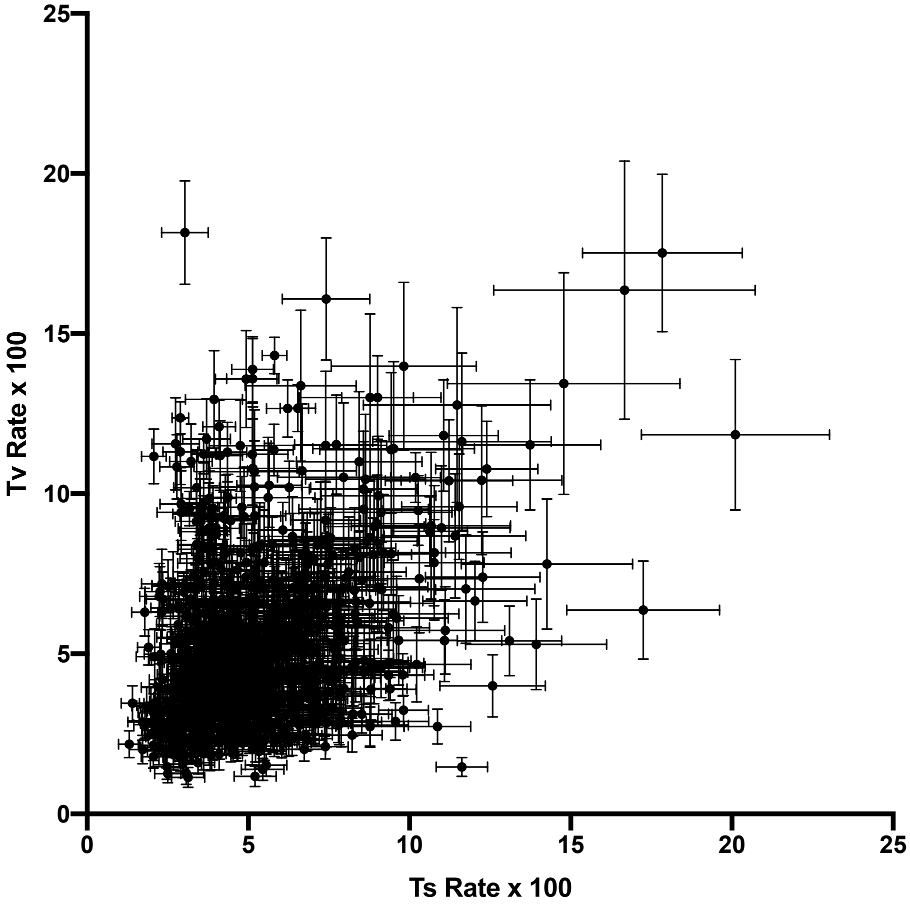

Figure Legend: Rates of substitution with 95% CI from T (a) and C (b) across hexanucleotide contexts with at least 50 transitions and 50 transversions.
