# Supplementary material for: Substitution rate heterogeneity across hexanucleotide contexts in noncoding chloroplast DNA
Source: G3 (Bethesda). 2022 Jun 14;12(8):jkac150. doi: 10.1093/g3journal/jkac150 (PMC9339276; doi:10.1093/g3journal/jkac150)
Supplement: jkac150_Supplementary_Figure_S2 [file jkac150_supplementary_figure_s2.pdf]

Figure S2

a)

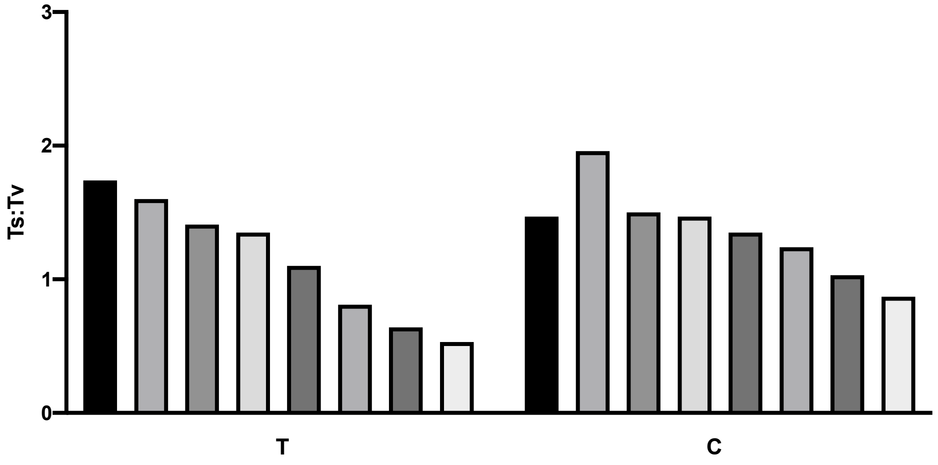

b)

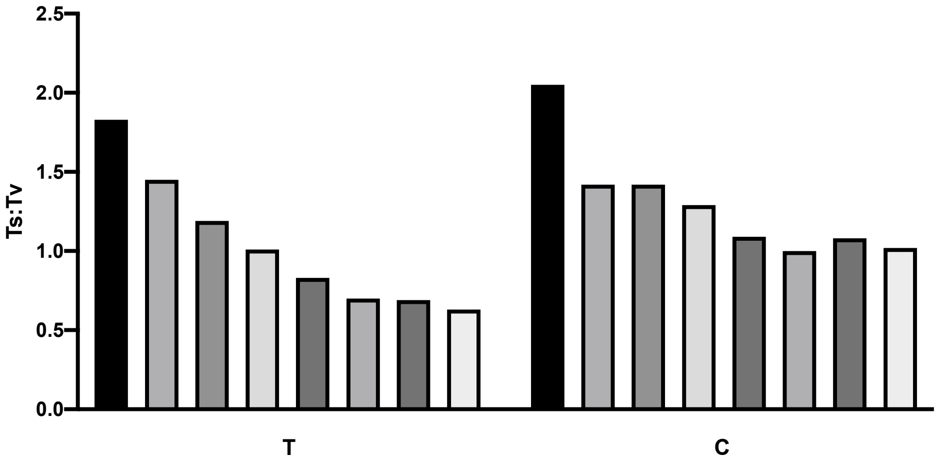

c)

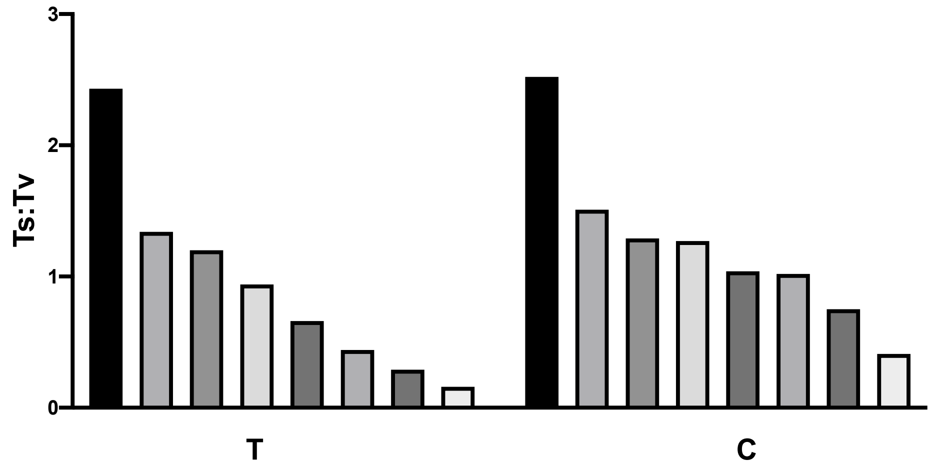

Figure Legend: Ts:Tv for substitutions of T and C relative to the ATI (a), RI (b), and the RATI (c) values of the hexanucleotide context. In each case the contexts are grouped into 8 categories with values of 0-3, 4-7, 8-11, 12-15, 16-19, 20-23, 24-27 and 28 from left to right.
