# Supplementary material for: Substitution rate heterogeneity across hexanucleotide contexts in noncoding chloroplast DNA
Source: G3 (Bethesda). 2022 Jun 14;12(8):jkac150. doi: 10.1093/g3journal/jkac150 (PMC9339276; doi:10.1093/g3journal/jkac150)
Supplement: jkac150_Supplementary_Figure_S3 [file jkac150_supplementary_figure_s3.pdf]

Figure S3

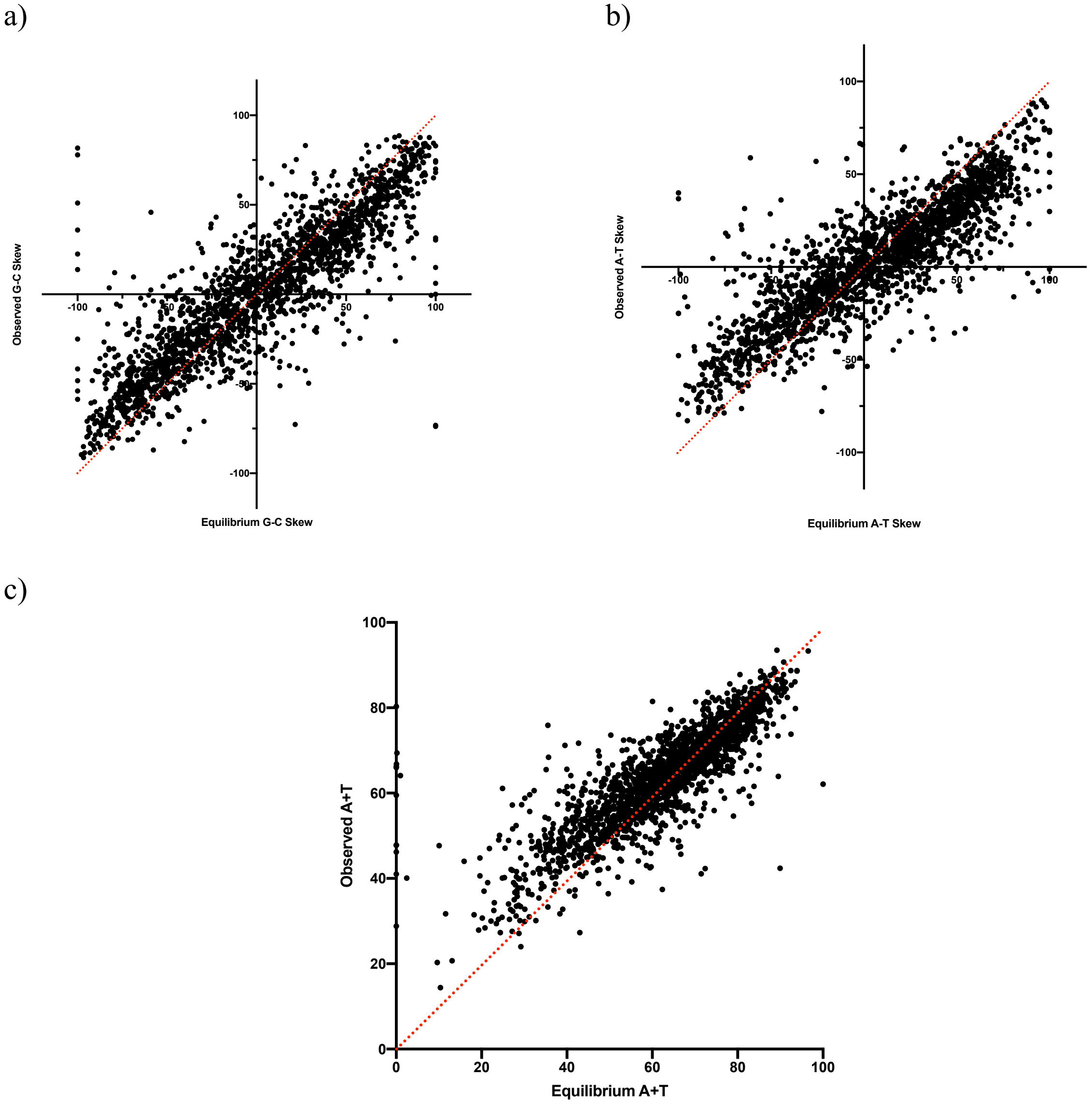

Figure Legend: For each context the stationary vector of the transition matrix was used to calculate predicted equilibrium base composition within that hexanucleotide. Compositions plotted are (a) A-T skew (b) G-C skew, and (c) A+T content. The equality line for each is shown in red.
